# A qualitative study of enablers and barriers to healthy eating in adults with Charcot Marie Tooth disease using the Theoretical Domains Framework and COM-B model

**DOI:** 10.1177/13591053261417031

**Published:** 2026-01-30

**Authors:** Petra Kolić, Jasmine Hearn, Matthew Jacques, Gita Ramdharry, Christopher I. Morse

**Affiliations:** 1 Manchester Metropolitan University Institute of Sport, UK; 2 Manchester Metropolitan University, UK; 3 University of Nottingham, UK; 4 UCLH NHS Trust, London, UK; 5 University College London, UK

**Keywords:** determinants of behaviour, Charcot-Marie-Tooth disease (CMT), qualitative research, interviews, behaviour change, healthy eating

## Abstract

Charcot-Marie-Tooth disease (CMT) is a progressive neurological disorder affecting the peripheral nervous system, with symptoms such as fatigue, pain and muscle weakness that can hinder engagement in health-promoting behaviours, including healthy eating. This study explored enablers and barriers to healthy eating among adults with CMT, using the Theoretical Domains Framework (TDF) and the COM-B model. Twenty-two semi-structured interviews were conducted with 17 women and 5 men (aged 25–73). Data were analysed using framework analysis. Identified barriers included symptom-related fatigue, particularly during periods of high demand (e.g. workdays), limited lifestyle-focused guidance in routine healthcare interactions and physical environmental constraints. Key enablers comprised meal planning, social support from family and online communities, recognition of the mental and physical benefits of healthy eating and access to resources that helped mitigate physical limitations. These findings provide a foundation for developing evidence-based, context-sensitive dietary interventions tailored to the lived experiences of individuals with CMT.

## Introduction

Charcot-Marie-Tooth disease (CMT) is a chronic, progressive neurological condition that affects the peripheral nervous system (Thomas et al., 2021). With an estimated incidence of 10–40 in 100,000 people (Okamoto and Takashima, 2023), it is the most frequently inherited neuropathy (Thomas et al., 2021). While disease onset usually occurs in the first two decades of life, with gradual progression over subsequent decades (Pareyson and Marchesi, 2009), CMT can affect individuals at any age (Okamoto and Takashima, 2023). Symptoms typically include distal muscle wasting and weakness in the distal segments of lower and upper extremities, foot arch and foot deformities, sensory loss in hands and feet, lessened tendon reflexes, pain and fatigue (Okamoto and Takashima, 2023; Pareyson and Marchesi, 2009). These symptoms can represent barriers to activities of daily life and can negatively impact quality of life (QoL; El-Abassi et al., 2014). For example, physical symptoms in lower extremities (e.g. ankle weakness), pose barriers to running, walking long distances, and moving on slippery ground (El-Abassi et al., 2014). Similarly, fatigue was reported to hinder daily tasks (e.g. walking, household duties) and social interactions (e.g. meeting friends outside one’s home) (Ramdharry et al., 2012). Negative implications for psychological well-being can arise through feelings of guilt and frustration (Ramdharry et al., 2012), embarrassment (McCorquodale et al., 2016), and body image concerns (Johnson et al., 2013) associated with the progressive and disabling nature of CMT. To address these barriers, interventions for health-promoting behaviours, for example, exercise, could be feasible, safe and produce short-term improvements in strength, aerobic capacity, and function among people with CMT (Conde et al., 2023). However, trials generally use variable protocols, short follow-up periods, and report adherence barriers (e.g. fatigue, pain), with long-term benefits and effects on other health-promoting behaviours remaining uncertain (Conde et al., 2023; Newman et al., 2023).

One area that has received limited attention is the association between CMT and diet with research in this field primarily focusing on nutritional supplementation (Burns et al., 2009) and intermittent fasting (Madorsky et al., 2009), which have only been evidenced to benefit individuals with CMT when combined with exercise (Smith et al., 2006). Research to date has overlooked the need for a healthy diet embedded within a healthy lifestyle to promote long-term well-being. This would be especially important considering co-morbidities, specific to CMT, that could show improvement from a healthy diet with a high prevalence of impaired glucose tolerance (Ursino et al., 2013), bone fractures (Pouwels et al., 2014) and obesity (Donlevy et al., 2021). At present, there are no specific nutritional guidelines for those living with CMT aside from general dietary guidelines (Pisciotta et al., 2021). In general terms, an integrated model of health is influenced by behaviours across the life span, including diet, and people who adopt healthy diets are more likely to be physically healthy and have improved QoL (Khaw et al., 2008).

Barriers to healthy eating are, however, likely to be accentuated in CMT through a combination of physical, psychological and social factors associated with the disease. For example, physical impairment in lower and upper extremities could pose a barrier to food shopping. High levels of pain and fatigue (Ribiere et al., 2012), and distal impairments (Eklund et al., 2009), along with concerns about limited control over one’s body and associated risk awareness (Price-Forde et al., 2025), for example, when using a sharp knife or carrying a heavy tray, may represent barriers to meal preparation. However, until these issues are examined and documented, it is impossible to develop evidence-based guidance. Understanding the multidimensional determinants of healthy eating (i.e. barriers and facilitators) represents an essential first step in producing dietary advice and interventions that support individuals living with CMT to implement sustainable healthy eating in everyday life (Cena and Calder, 2020).

The COM-B model is an established approach used to identify determinants of behaviour (Michie et al., 2014). It incorporates a person’s capability (the capacity to engage in behaviour), opportunity (environmental factors and resources that influence behaviour), and motivation (a person’s inclination to engage in behaviour) with six sub-domains (two per domain) that capture factors known to influence the adoption of new behaviour. These encompass (i) psychological and (ii) physical capability, (iii) physical and (iv) social opportunity, (v) reflective and (vi) automatic motivation (Michie et al., 2014). The Theoretical Domains Framework (TDF) builds on the COM-B model, rationalising 33 psychological theories to offer a comprehensive framework for identifying determinants of behaviour (Atkins et al., 2017). The TDF captures multidimensional domains that can act as enablers of, and barriers to, engaging in a behaviour (e.g. healthy eating) and can be mapped onto the COM-B model. The TDF domains include ‘knowledge’, ‘memory, attention, and decision processes’, ‘social, emotional, and intellectual understanding’, ‘behaviour regulation’ and ‘cognitive and interpersonal skills’ (psychological capability); ‘physical skills’ and ‘physical capacity’ (physical capability); ‘environmental context and resources’ (physical opportunity); ‘social influences’, ‘reinforcement’ and ‘beliefs about consequences’ (social opportunity); ‘social and professional role and identity’, ‘beliefs about capabilities’, ‘beliefs about consequences’, ‘optimism’, ‘intentions’ and ‘goals’ (reflective motivation); and ‘emotion’ (automatic motivation). A framework approach (Gale et al., 2013), where behavioural determinants are identified and then mapped onto the TDF and COM-B model is particularly useful to facilitate the identification of behavioural determinants to consider as part of behaviour change interventions, and was therefore adopted in this study (Michie et al., 2014). The TDF and COM-B model have been used successfully to shape behaviour change interventions in other clinical settings, including weight loss in obese adults (Coupe et al., 2022), pain medication adherence in adults with chronic pain (Timmerman et al., 2017) and decision-making in rheumatoid arthritis (Barber et al., 2021). To our knowledge, however, they have not been implemented within the context of diet in CMT. Therefore, as the first stage of intervention development, the aim of this study was to identify enablers and barriers which motivated and prevented healthy eating in adults with CMT. The following research questions were asked:

- How do adults with CMT experience and describe the enablers and barriers that motivate or prevent them from engaging in healthy eating?
- How do the identified enablers and barriers to healthy eating among adults with CMT map onto the TDF and COM-B model?

## Material and methods

### Research positioning

This study was conducted by a multidisciplinary team. The lead researcher (first author), a social scientist with 8 years of qualitative research experience at the time of the study, was supported by a health psychology researcher with expertise in behaviour change (second author), two clinical physiologists with expertise in neuromuscular diseases (third and fifth author) and an allied health professional in neuromuscular diseases (fourth author). To accommodate our diverse research approaches, we adopted a pragmatic research positioning. Pragmatism recognises the dynamic interplay between knowledge, experience and action and prioritises real-world problem-solving (Morgan, 2014). In this view, knowledge is evolving, tentative, and situated within people’s interpretations of their experiences (Rosiek, 2013). We therefore considered our research an active process of meaning-making that was co-created with and situated within the lives of the participants and the research team (Morgan, 2014). These assumptions shaped our decision to engage adults with CMT through qualitative inquiry to facilitate in-depth understandings of their perceptions, interpretations and lived realities with attention to how past and present experiences were shaped by future possibilities and, equally, how future possibilities may shape these experiences (Rosiek, 2013). We therefore explored not only *how* adults with CMT perceived enablers and barriers to healthy eating, but also *why* certain enablers and barriers to healthy eating were meaningful to them (Morgan, 2014). The TDF and COM-B model served as a theoretical lens to generate knowledge that is conceptually rigorous and practically applicable, along which using a framework approach enabled us to integrate participant perspectives within the COM-B model to ensure relevance of insights for future interventions (Gale et al., 2013).

### Participant recruitment

Following ethical approval from the Faculty of Science and Engineering Ethics Committee at Manchester Metropolitan University (approval no. 48048), an email invitation was sent to 37 individuals who had participated in previous research conducted by the research team and had agreed to be contacted about further study participation. Participants were purposefully sampled to generate information-rich knowledge central to the inquiry, including adults aged 18 years or older who were diagnosed with CMT. The final sample comprised 22 participants (aged 25–73 years), including 17 women and 5 men (see Supplemental File 1 for detailed participant characteristics). All participants gave written consent before study participation, for the purpose of which the first author emailed a consent form template to participants, who signed and returned the form via email to her prior to data collection.

### Data collection

In keeping with our pragmatic research positioning, we utilised semi-structured interviews to develop rich knowledge of enablers and barriers to healthy eating in adults with CMT. Semi-structured interviews were useful to stimulate conversations that were relevant to the inquiry, while allowing participants to discuss experiences that were individually meaningful to them (Brinkmann and Kvale, 2018).

Before the interviews, we developed an interview guide outlining example questions, which was informed by theoretical concepts, empirical literature, and critical conversations within the research team (Brinkmann and Kvale, 2018). Each interview started with an introduction, a summary of the purpose of the interview and demographic questions (age, ethnicity). The conversation then explored enablers and barriers to healthy eating associated with CMT using open-ended questions in relation to the TDF domains outlined in Atkins et al. (2017). Questions sought to explore *what* the participants’ experiences were, *how* the participants navigated healthy eating, and *why* certain factors acted as enablers and barriers to healthy eating. For example, to explore the TDF domain ‘knowledge’, we asked questions including ‘What do you know about healthy eating in CMT? Its importance, value, benefits etc.’ as well as ‘How have you come to learn this knowledge?’ and ‘How and why, in certain ways, does this knowledge affect your eating habits?’ Although the interview guide was useful to steer conversations to topics relevant to the inquiry, the interviewer was attentive to the participants’ unique narratives and tailored the order in which questions were asked to how individual conversations unfolded. The interviewer utilised curiosity-driven and probing questions (e.g. ‘Can you give me an example?’, ‘How does this make you feel?’) to elicit in-depth responses about what was important to participants, including determinants of behaviour that may not be identified as part of the TDF and COM-B model (McGowan et al., 2020).

Each participant took part in one interview, with all interviews conducted by the first author via video call using Microsoft Teams. The decision to conduct interviews remotely served to remove potential barriers arising from travel requirements of in-person interviews (O’Connor and Magde, 2017). Approximately 7 days before each interview, the first author emailed the broad topics to be discussed in the interview to participants to help reduce interview anxiety and allow time for reflection, particularly surrounding eating habits, practical challenges in daily life and meanings of healthy eating (Haukås and Tishakov, 2024). In total, 22 semi-structured interviews were conducted, amounting to a total of 13 hours of interview data (30–50 minutes per interview). Interviews were audio-recorded and transcribed by the first author. Pseudonyms have been used throughout this manuscript to ensure the anonymity of participants.

### Data analysis

The data analysis followed a framework approach (Gale et al., 2013). This began with attentive (re)reading of interview transcripts and line-by-line coding of transcribed data to define descriptive codes and categories that encapsulated key data extracts (Gale et al., 2013; McGowan et al., 2020). Following this inductive analysis, the analysis became more deductive, where broader categories were mapped onto the domains of the TDF and then, directly onto components of the COM-B model (Gale et al., 2013). After the first author completed a preliminary analysis, she worked closely with the second author to reflect upon initial interpretations, revise codes, merge smaller codes where appropriate, group codes into broader categories and develop context-sensitive insights with relevance for academic understanding and practical application (Bingham and Witkowsky, 2022). Finally, the other members of the research team helped review, discuss, and revise interpretations.

Integrating inductive and deductive analysis was useful to ensure rigour (Bingham and Witkowsky, 2022). While descriptive codes generated during the inductive data analysis were useful to understand participant perspectives without forcing the data into predetermined theory and potentially missing factors outside of the TDF and COM-B model, the subsequent deductive analysis facilitated a focused approach to understanding how specific factors identified in the TDF and COM-B model might act as enablers of and barriers to healthy eating (Atkins et al., 2017; McGowan et al., 2020).

## Results

We identified five of the six categories of the COM-B model as enablers of, and barriers to, healthy eating in adults with CMT, including: reflective motivation, physical capability, physical opportunity, psychological capability and social opportunity (automatic motivation was not reflected in the data). The following pages are structured using the COM-B categories (generated through deductive analysis), in an order which we believe offers the most coherent narrative. We signpost to TDF domains where we present results that align with them. To exemplify participant perspectives, we present data samples within each of the COM-B categories. We explicitly note themes, which were generated through inductive analysis and associated data samples (including some that extend beyond those presented in the main manuscript), in Supplemental File 2.

### Reflective motivation

Participants recognised the importance of healthy eating, and many demonstrated an understanding of its benefits to physical health and well-being (TDF domains: ‘beliefs about capabilities’, ‘beliefs about consequences’, ‘intentions’, ‘goals’ and ‘optimism’):

When I eat better, I have more energy to do things. (Grace)

Well, it’s my health. I can keep control of diet and keep at bay diabetes, cholesterol, heart disease. (Shelley)

Healthy eating is the best way to minimise the adverse consequences of unhealthy eating. I don’t want to get diabetes. I don’t want to get fatty liver disease, and eating healthy will give me the best chance of living healthily and sustaining my well-being. (Jim)

The participants also reflected on the benefits of healthy eating to their mental well-being, describing themselves as happy and more content (TDF domains: ‘beliefs about consequences’ and ‘optimism’) when eating healthy foods:

Mentally, it makes me feel better. Knowing I’m making good choices makes me happier. (Vera)

I’m feeling better about myself, mentally. This might seem shallow, but when you look better physically, you feel better, your mental health is better. (Tamsin)

When discussing the benefits of healthy eating, participants spoke of the importance of regulating their food intake for weight management purposes, through strategies, such as intermittent fasting (TDF: ‘beliefs about capabilities’, ‘intentions’ and ‘goals’). Participants felt under pressure to eat healthily and expressed weight loss concerns, as many knew that a healthy weight was important to help minimise the impact of CMT symptoms on their ability to engage in daily activities (e.g. walking up and down stairs). Maintaining a healthy weight was also a concern because if participants were to gain weight, the only way to lose this extra weight would be to reduce food intake, as increasing energy expenditure (through physical activity) was often no longer an option due to the severity of their CMT symptoms (TDF domain: ‘beliefs about consequences’). In the participants’ words:

I don’t exercise, I’m not burning calories anymore. I’ve got weakness in my legs, so it’s not good to put on weight. So, I must keep my weight down and eat as healthily as I can. (Drake)

I would eat breakfast, but I try to keep my weight down and breakfast is the meal I miss the least. I can’t exercise anymore, so that’s my only option. (Flora)

An important factor that could act as an enabler or barrier to healthy eating represented the participants’ mindset, their thoughts and feelings (TDF domains: ‘beliefs about capabilities’, ‘intentions’):

Part of healthy eating is your psyche and your motivation. If I didn’t have the will to cook, I would find it really distressing. (Harper)

It depends, what mindset I’m, whether I self-sabotage, which I do a lot. If something knocks me off, that can be it for weeks. (Joy)

### Physical capability

The physical symptoms of CMT represented one of the most significant barriers to healthy eating (TDF domain: ‘physical skills’). Commonly reported physical symptoms that led participants to avoid activities associated with healthy eating (e.g. cooking from scratch) or adapt what they did (e.g. using kitchen gadgets) included fatigue, pain, poor strength (in hands and/or legs) and issues with precision and fine motor skills. The following quotes exemplify the participants’ experiences:

The physicality of food preparation. Chopping can be precarious, because it’s a lot easier to chop my finger and carrying large pans of water for boiling. If my strength is not there, then that can be dangerous. (Grace)

Standing. I can’t stand and prepare food for more than an hour. At the supermarket, I wear gloves, because it is so cold, I lose sensation in my fingers. (Harper)

A recurring discussion point represented the budgeting of energy (TDF domain: ‘physical capacity’) to be able to engage in activities associated with healthy eating (e.g. food shopping, cooking, eating, washing up, tidying). This was necessary because fatigue represented one of the greatest barriers to healthy eating. Participants addressed the need to anticipate fatigue levels in their plans of what (and how much) they could do in any given day, which in turn could affect their ability to engage in healthy eating. The following data extracts exemplify the participants’ views:

You have to factor in how tired you get and doing one job can throw you for the rest of the day. If you’re having a bad day, but it needs doing, then you’d only have so much energy to do everything else. It’s difficult. You need to think, “How much energy is this going to take me and how tired am I going to be?” It’s a rubbish way to live. (Vera)

It’s always managing energy levels. Doing what everybody else does normally in a day will drain my battery quicker. I have to budget my energy to make sure that I can finish what I start. (Drake)

### Physical opportunity

All participants identified their physical environments (resources and facilities), as barriers to healthy eating. This included food shopping (travelling to the supermarket, walking, filling and emptying a trolley, transporting and tidying away purchased food at home) through to meal preparation, eating, washing dishes and tidying the kitchen. However, all participants also described a variety of coping strategies to overcome these barriers (TDF domains: ‘environmental context’ and ‘resources’). Many participants used kitchen gadgets and utensils to help them prepare healthy food, had made house adaptations to facilitate moving around their home more comfortably or relied on a car to travel to the supermarket:

My hands are weak and they’re a bit flawed. I have everything possible that can help me. I have food processers, spiralizers, things to open cans for me, things to beat things with. And I have everything nearby, so it’s easy to use. (Kitty)

I have a seat in the kitchen. And that makes me more likely to cook from fresh. I sit on it and take my time. (Beck)

We recently got a new kitchen, and we deliberately designed it knowing that I would be less physically capable. (Anni)

I have a car, it’s automatic. I can only drive automatic. I have a three-year licence, and I use it all the time. (Juliet)

A car to travel to the supermarket was particularly important as many participants, while acknowledging the benefits of online food shopping in principle, found that having food delivered to the house was difficult, as participants were not fully in control of this process. Particularly problematic was the arrival of goods in large baskets placed on the ground, which was challenging, for some impossible, to pick up and carry into the house. It put participants under time pressure to tidy away items as quickly as possible. Emmeline and Vera explained:

They deliver huge baskets, and you’ve got to bend down. But I can only carry a couple of items at a time because of my balance, so it takes twice as long to do that. (Emmeline)

Trying to get shopping from the door into the kitchen. I know I should use online shopping more, but the number of times I’ve done it, and they’ve been out of stock of something, and substituted something quite major for something else, or the shelf life is bad. And once it’s at my house, I find it hard to tidy it away quickly. (Vera)

In the context of their physical environment, participants reflected on their own privilege, acknowledging how challenging it must be for others with fewer (financial) resources to buy fresh food and prepare healthy meals:

Cost is a huge thing, especially for disabled people and I recognise my privilege that I’ve got disposable income that I can choose to spend on food. (Tamsin)

Healthy food can be more expensive. Same with having fresh vegetables, salmon, and good quality meat. There’s a cost associated with that. It’s cheaper to eat less healthy food, generally. Having fresh fruit in the house all the time, that’s a privilege. We’re in a very luxurious position that I don’t have to think about our food budget. So, it’s not a barrier to me, but it is globally a barrier. (Kendra)

### Psychological capability

All participants suggested that healthy eating comprised of eating whole foods, with many highlighting the importance of a balanced diet that included different types of foods, all within reason (TDF domains: ‘knowledge’, ‘behaviour regulation’ and ‘cognitive skills’). The following quotes exemplify what participants said:

Healthy eating is having a balance, knowing that you can enjoy the richer foods, foods that one would consider less good for you, sensibly of course. (Letty)

I’m very aware there’s no one special diet for CMT. It’s just that boring, but healthy, balanced diet, you know, making sure you’re getting a good spread of the things you need. (Kendra)

Planning and being organised were important enablers to implement healthy eating in daily life (TDF domains: ‘knowledge’, ‘memory, attention and decision processes’, and ‘social, emotional, and intellectual understanding’). An organised approach to buying and preparing healthy food required thinking ahead of time and consideration of factors that might represent barriers to healthy eating:

I plan ahead. The challenge for us is that my husband doesn’t like cheese, so we use pulses, beans. I’ll sometimes make lasagna and use lentils in the sauce and avoid putting cheese on top of it. I always have to make sure I have ingredients in the house to make those alternatives. (Harper)

While planning and organisation were helpful to support healthy eating, the participants acknowledged several circumstances that represented barriers to adhering to healthy eating habits (TDF domain: ‘behavioural regulation’). For participants in the workforce, navigating healthy eating around work and CMT symptoms was difficult:

Workdays are more challenging because I don’t work from home. On workdays, I can’t go into my kitchen and make something, like a salad, so I grab a sandwich normally, so I don’t have to prepare anything, which doesn’t have the vegetables in it that I would have in a salad. (Rosie)

I aim to cook from scratch when I finish work, but sometimes can’t be bothered. So, I throw things in the oven or have pot noodles. I find it easier at the weekend. And I don’t work Thursday. On a Thursday, I think, “I’ll make something from scratch” because that’ll be my one thing for the day. (Beck)

I have my full-time days at the end of the week, so Wednesday, Thursday, Friday, it really starts dipping back to convenience foods. (Anni)

For those who were retired, planning revolved around when to eat and deciding when to resist food (e.g. to avoid eating out of boredom and risk gaining weight):

Since I retired, I’ve got more time to think about eating. Which is good and bad. If I eat breakfast, it means I can’t eat any lunch because I don’t want to eat too much. So, it’s constantly weighing up my options. (Emmeline)

I try to manage what I eat, but I find that hard because of the snacking. It takes a lot of discipline. When I was working, it was much easier. I would take lunch to work. I didn’t snack. (Letty)

### Social opportunity

When discussing pathways of diagnosis and care, most participants identified a perceived lack of consideration and support for healthy eating in their interactions with healthcare professionals (TDF domains: ‘social influences’, ‘reinforcement’ and ‘emotion’). In the participants’ own words:

Absolutely zero. I was in a teaching hospital, and they were kind but all they said was, “You’ve got this, and there’s no cure.” Every time I go for a check-up, which is once a year, they are nice. It’s the only time given me to talk about my symptoms and then they say, “Yes, this is normal”. But apart from it, nothing. (Kim)

It was just a case of “This is what you have, goodbye”. In my checkups with the neurologist now, no one has ever mentioned eating. (Emmeline)

Only Vera had received tips from her consultant on how to make changes to food preparation routines to accommodate the needs and constraints arising from CMT. Vera’s experience demonstrated how the sharing of knowledge and ideas could have a positive impact on healthy eating:

I’ve had advice from the consultant. When I’m doing potatoes in a pan, because I’m cooking for a lot of us that means I need a big pan. And she suggested, “Why don’t you put two smaller pans on?” Little things can make a huge difference. Or instead of straining the water out, use a spoon with holes to scoop out the potatoes. And you know what, those tips make a real positive impact. I still manage making nutritious healthy foods for the family. (Vera)

The overall lack of acknowledgement of healthy eating by healthcare professionals could explain the participants’ drive to learn about CMT through self-directed learning. The participants relied on online platforms, social media communities and the work of charities (e.g. CMT UK) to better understand the realities of living with CMT and what implications this may have for activities of daily life, including healthy eating:

The best thing about having CMT is the strong community. It encourages each other to be active and eat healthily. People need it. (Kendra)

I joined this support group in the West Midlands, they make me feel that help is out there. (Gillian)

It became easier when you could get information online. And when there were patient groups online. Because there’s no cure, a lot of the things with CMT are practical things you can do to make life easier, and the best people to get advice on that, are people who are facing the same problems. You tend to see a doctor once a year, and they’re busy, so you don’t feel like you can ask many questions. (Flora)

An important enabler to healthy eating was the immediate social environment, in particular, spouses and partners with whom the participants lived. For many, such as Emmeline and Becky, it was helpful to be able to rely on them to complete tasks associated with healthy eating and overcome barriers arising from the physical symptoms of CMT (discussed in the earlier section on physical capability):

The preparation is a problem in my case, which is why my husband tends to cook. (Emmeline)

My husband can chop things for me. If he’s around, he’ll do it. (Beck)

## Discussion

This qualitative study utilised the TDF and COM-B model to offer a theoretical interpretation of the behavioural determinants associated with healthy eating in adults with CMT (Michie et al., 2014). We mapped enablers and barriers to healthy eating onto COM-B sub-domains, including reflective motivation, physical capability, physical opportunity, psychological capability and social opportunity (Supplemental File 2).

Knowledge of healthy eating (COM-B sub-domain: psychological capability) represented an important enabler, with participants overall defining a healthy diet as characterised by eating whole foods and home-cooked meals with enjoyment of indulgent and, ultimately, ultra-processed foods on rare occasions. Eating a range of foods was seen to nourish the body and have a positive impact on health outcomes (Cena and Calder, 2020; de Ridder et al., 2017). Especially with the benefits to physical and mental health in mind, the participants recognised the importance of healthy eating (COM-B sub-domain: reflective motivation). Similar to findings from research investigating the impact of diet and food choices on cognitive function (Strasser and Fuchs, 2015), fatigue (Gifkins et al., 2018), anxiety and depression (Saul et al., 2022), the participants in our study felt that eating a balanced diet led them to feel better, more energised and content. In contrast to the unpredictable pace at which CMT progressed and could, in turn, negatively affect independence, participants emphasised that their diet represented something that they were able to control (Haahr et al., 2011), supporting feelings of autonomy and agency.

To act upon the knowledge and importance attached to healthy eating, important routines represented being organised, planning meals and preparing foods ahead of time (COM-B sub-domain: psychological capability; de Ridder et al., 2017; Haahr et al., 2011; Ramdharry et al., 2012). However, participants acknowledged that even though planning, organisation, and preparation could act as enablers of healthy eating, they were difficult to maintain during busy periods, for example on workdays, when participants needed to navigate numerous activities and the stressors arising from them in 1 day (e.g. being at work, then doing the school run, and then cooking dinner), and consequently felt more fatigued (Ramdharry et al., 2012). Fatigue represented the most impactful physical barrier to healthy eating, in addition to which participants also discussed reduced fine motor skills (Eklund et al., 2009), sensory loss (Pareyson and Marchesi, 2009), pain (El-Abassi et al., 2014) and lack of muscle strength (Okamoto and Takashima, 2023), predominantly in lower and upper extremities (COM-B sub-domain: physical capability). Participants identified the budgeting of energy as a fatigue management strategy by trying to anticipate how tiring daily tasks would be to ensure they could complete everything they intended to do (Audulv et al., 2021). Having to predict how fatigue might impact what could be achieved each day was emotionally and intellectually demanding (Pearson et al., 2022) and could negatively affect the participants’ mindset (COM-B sub-domain: psychological capability) away from prioritising healthy eating towards eating convenience foods (e.g. crisps, biscuits) that rendered an instant ‘boost’ of energy and sense of fulfilment.

With CMT being a progressive disease that affected the lower extremities, participants felt under pressure to eat healthily to manage their body weight (COM-B sub-domain: reflective motivation) as they were aware that weighing more could hinder completion of daily tasks (e.g. walking). Participants were mindful that if (or when) their CMT progressed to an extent that meant they were no longer able to expend calories by being physically active, it was essential to manage body weight by regulating food (i.e. calorie) intake to avoid gaining weight that could then be difficult to lose. This was particularly addressed by participants, who were retired and described that their food planning revolved around what, when and how much to eat. While retired participants no longer needed to plan healthy meals for work (as described by those in the workforce), retirement led to more time spent in the home environment, where a choice of (unhealthy) foods was readily available. Similar to the impact of fatigue, it was cognitively demanding for participants to exert self-control and eat healthily (COM-B sub-domain: psychological capability) when competing cues could divert participants away from healthy eating (e.g. boredom could be misinterpreted as hunger; McCarthy et al., 2017).

Interactions with others could act as enablers and barriers to healthy eating (COM-B sub-domain: social opportunity). The participants suggested that healthcare professionals offered little to no advice to help navigate lifestyle behaviours, such as healthy eating, during CMT diagnosis and review appointments (Ramdharry et al., 2012). The oftentimes annual medical reviews of CMT, focused on symptom severity and progression, as opposed to the whole person (Bellass et al., 2024), with only one participant in our study reporting that her consultant had discussed strategies to facilitate meal preparation and cooking. This limited consideration by healthcare professionals of healthy eating could stem from a combination of factors, including limited time during appointments (Bellass et al., 2024) and limited knowledge of what CMT patients wanted or needed from review appointments (Rule et al., 2024). Our participants therefore relied on other social environments (COM-B sub-domain: social opportunity), with many highlighting the value of support groups, charities and online communities (e.g. social media), to develop self-management strategies that facilitated healthy eating and, more broadly, to better cope with the practical and emotional challenges that living with a progressive disease, like CMT, entailed (Audulv et al., 2021; Reeves et al., 2014). Sharing experiences with others who had CMT rendered a sense of belonging, helped reduce feelings of loneliness and frustration associated with the daily struggles of CMT and formed opportunities to learn about ways to resolve these struggles (Reeves et al., 2014). Some of our study participants even suggested that people who lived with CMT were best placed to share knowledge and adaptations to navigate daily life, as they had first-hand experience of CMT.

An important enabling factor in the participants’ immediate social environments represented spouses, partners, and family members (COM-B sub-domain: social opportunity). In fact, most participants spoke of adult family members who lived in their home (e.g. husband, wife, son) who took an active role in supporting healthy eating tasks (Carroll et al., 2021), especially when they themselves were no longer able to complete these because of their CMT progression (e.g. help with chopping due to limited fine motor skills in hands). It could be suggested that this daily support, in combination with support from (online) groups, positively impacted our participants’ perceived ability to cope with the progressive nature of CMT and, in turn, their life satisfaction (Şahin et al., 2019).

Social support networks were important to help navigate physical environments, including spaces (e.g. supermarkets) and processes (e.g. grocery delivery), that were not designed with physically disabled people’s needs in mind (COM-B sub-domain: physical environment) and therefore posed barriers to completing healthy eating tasks (e.g. food shopping)(Rule et al., 2024). However, our participants believed they were privileged to have sufficient financial resources, strong support networks, and the ability to seek out new knowledge (through internet access or knowledge of how to navigate websites), which enabled them to afford and access fresh food, gadgets, and adaptations in the home that facilitated healthy eating despite barriers in their physical environments (Audulv et al., 2021). Participants were aware of how experiencing an intersection of barriers (e.g. arising from physical disability, socio-demographic characteristics, employment status, or poor transport) could hinder equitable access to healthy eating (Wolbring and Deloria, 2024) and put them at an increased risk of suffering from the adverse effects of co-morbidities specific to CMT, (e.g. obesity – Donlevy et al., 2021), negatively impacting health and well-being.

It is noteworthy that our participant sample was relatively small and homogenous, with a prevalence of female participants who had good support and resources in place. As a result, we did not capture the potential influence of lower socio-economic status or diverse cultural, religious, and ethnic backgrounds. Neither was there scope to unpack whether gender-specific factors shaped how determinants of healthy eating were perceived. Future research should therefore prioritise capturing larger groups of participants from varied socio-demographic groups to better understand behavioural determinants of healthy eating that reflect the diversity among populations in of high-income countries, such as the UK. This work could also include a focus on how stereotypical gender norms and divisions of responsibility in household and family life may shape behaviours associated with healthy eating in CMT. This could be accomplished via mixed-method approaches that, for example, incorporate large-scale quantitative surveys with qualitative interviews or focus groups. We moreover suggest that it is important to identify determinants of healthy eating in CMT across the lifespan. This includes examining age-specific enablers and barriers (e.g. adolescence vs early adulthood) and differences between those newly diagnosed and long-term CMT patients. Understanding how behavioural determinants evolve with age and disease progression can support the development of inclusive, transferable interventions tailored to various life stages and realities of living with CMT.

### Applied implications

To support the translation of study results into practice, key stakeholders (people with CMT, healthcare professionals, advocacy groups) should be at the centre of intervention design, implementation and evaluation, in addition to which established frameworks for the (re)design of behaviour change interventions (e.g. APEASE criteria: Acceptability, Practicability, Effectiveness, Affordability, Side-effects, and Equity) could support sustainable improvement of CMT management, support, and care (Michie et al., 2013, 2014). We offer ideas for intervention development:

- Accessible information about healthy eating: Given the variability in onset and progression of CMT, evidence-based healthy eating information should be provided in accessible formats tailored to different ages and disease stages (e.g. digital booklets, leaflets at medical appointments). Integrating this advice with physical activity recommendations (Smith et al., 2006) could further support independence, health and confidence in maintaining a healthy lifestyle with CMT
- Support for the physical aspects of healthy eating: Our study participants identified physical CMT symptoms and physical environments (e.g. supermarket) as significant barriers to completing tasks associated with healthy eating. Initiatives, such as dedicated shopping times for people with disabilities or elevated baskets when food is delivered to people’s homes, could represent effective, positive changes to support healthy eating.
- Awareness and understanding: Our study highlights interactions with healthcare professionals during CMT-related appointments as key opportunities to adopt a ‘whole-person’ approach that considers individual lifestyles, behaviours and needs. While it would be beneficial for healthcare professionals to have more knowledge of CMT, we agree with previous work (e.g. Mudge et al., 2016), that listening to patients and drawing on their lived experiences may be more valuable than specialist expertise alone. Interventions that strengthen the support networks identified by our participants could help with the dissemination of effective self-management strategies (e.g. healthy eating practices) within the CMT community, enhancing disease awareness and understanding among both patients and healthcare professionals (Plackowski and Bogart, 2023).

## Conclusion

This qualitative study identified enablers and barriers to healthy eating in adults with CMT using established behaviour change theory for intervention design (TDF and COM-B model). The results offer insight into the physical, psychological, and social determinants of behaviour associated with healthy eating and represent the necessary first step in the development of evidence-based interventions aimed at supporting healthy eating in CMT. Developing interventions that consider multifactorial determinants of healthy eating will offer greater potential to positively impact the QoL, long-term health and well-being of individuals living with CMT.

## Supplemental Material

sj-docx-1-hpq-10.1177_13591053261417031 – Supplemental material for A qualitative study of enablers and barriers to healthy eating in adults with Charcot Marie Tooth disease using the Theoretical Domains Framework and COM-B model Supplemental material, sj-docx-1-hpq-10.1177_13591053261417031 for A qualitative study of enablers and barriers to healthy eating in adults with Charcot Marie Tooth disease using the Theoretical Domains Framework and COM-B model by Petra Kolić, Jasmine Hearn, Matthew Jacques, Gita Ramdharry and Christopher I. Morse in Journal of Health Psychology

sj-docx-2-hpq-10.1177_13591053261417031 – Supplemental material for A qualitative study of enablers and barriers to healthy eating in adults with Charcot Marie Tooth disease using the Theoretical Domains Framework and COM-B model Supplemental material, sj-docx-2-hpq-10.1177_13591053261417031 for A qualitative study of enablers and barriers to healthy eating in adults with Charcot Marie Tooth disease using the Theoretical Domains Framework and COM-B model by Petra Kolić, Jasmine Hearn, Matthew Jacques, Gita Ramdharry and Christopher I. Morse in Journal of Health Psychology
