# Supplementary material for: A qualitative study of enablers and barriers to healthy eating in adults with Charcot Marie Tooth disease using the Theoretical Domains Framework and COM-B model
Source: J Health Psychol. 2026 Jan 30;31(11):4392–406. doi: 10.1177/13591053261417031 (PMC13490675; doi:10.1177/13591053261417031)
Supplement: Supplementary file 1 [file sj-docx-1-hpq-10.1177_13591053261417031.docx]

**Supplementary file 1. Characteristics of participants in the study titled ‘A qualitative study of enablers and barriers to healthy eating in adults with Charcot Marie Tooth disease using the Theoretical Domains Framework and COM-B model’**

| **Pseudonym** | **Gender** | **Age** | **CMT type** | **Age at diagnosis** | **Years since diagnosis** |
| --- | --- | --- | --- | --- | --- |
| Harper | F | 64 | 1A | 64 | 0 |
| Vera | F | 39 | 1A | 12 | 27 |
| Kendra | F | 42 | 1A | 37 | 5 |
| Grace | F | 25 | 1A | 22 | 3 |
| Joy | F | 61 | 1A | 46 | 15 |
| Kim | F | 55 | 1A | 52 | 3 |
| Emmeline | F | 62 | 1A | 57 | 5 |
| Anni | F | 46 | 1A | 31 | 15 |
| Rosie | F | 31 | 1A | 20 | 11 |
| Juliet | F | 62 | 1A | 55 | 7 |
| Flora | F | 59 | 1A | 30 | 29 |
| Jim | M | 73 | 1A | 65 | 8 |
| Dean | M | 38 | 1A | 33 | 5 |
| Drake | M | 45 | 1A | 28 | 17 |
| Letty | F | 73 | 2A | 67 | 6 |
| Shelley | F | 49 | Confirmed diagnosis, but unsure of CMT type | 47 | 2 |
| Tamsin | F | 40 | Confirmed diagnosis, but unsure of CMT type | 1 | 39 |
| Kitty | F | 69 | Confirmed diagnosis, but unsure of CMT type | 32 | 37 |
| Beck | F | 35 | Confirmed diagnosis, but unsure of CMT type | 5 | 30 |
| Gillian | F | 74 | Confirmed diagnosis, but unsure of CMT type | 66 | 8 |
| Toby | M | 63 | Confirmed diagnosis, but unsure of CMT type | 18 | 45 |
| Damon | M | 55 | Confirmed diagnosis, but unsure of CMT type | 55 | 0 |
