# Supplementary material for: A qualitative study of enablers and barriers to healthy eating in adults with Charcot Marie Tooth disease using the Theoretical Domains Framework and COM-B model
Source: J Health Psychol. 2026 Jan 30;31(11):4392–406. doi: 10.1177/13591053261417031 (PMC13490675; doi:10.1177/13591053261417031)
Supplement: Supplementary file 2 [file sj-docx-2-hpq-10.1177_13591053261417031.docx]

**Supplementary file 2. Data samples organised by themes, TDF and COM-B domains to support the manuscript ‘A qualitative study of enablers and barriers to healthy eating in adults with Charcot Marie Tooth disease using the Theoretical Domains Framework and COM-B model’**

Note: Pseudonyms have been used throughout this document

| **COM-B domain** | **TDF domains** | **Themes with data samples** |
| --- | --- | --- |
| Reflective motivation | Beliefs about capabilities  Beliefs about consequences  Intentions  Goals  Optimism | **Importance of healthy eating**  *When I eat better, I have more energy to do things. (Grace)*  *Well, it's my health. I can keep control of diet and keep at bay diabetes, cholesterol, heart disease. (Shelley)*  *Healthy eating is the best way to minimise the adverse consequences of unhealthy eating. I don't want to get diabetes. I don't want to get fatty liver disease, and eating healthy will give me the best chance of living healthily and sustaining my well-being. (Jim)*  *Mentally it makes me feel better. Knowing I’m making good choices makes me happier. (Vera)*  *I'm feeling better about myself, mentally. This might seem shallow, but when you look better physically, you feel better, your mental health is better. (Tamsin)*  *I feel better if I have a lot of vitamins, so I take vitamins, but I also eat mushrooms, eggs, because that gives me a natural boost. I eat a lot of peppers and stuff that has a lot of vitamin C, because as I get tired, my immune system dips. I also have magnesium, because magnesium stops cramps. For every meal apart from breakfast, I have some salad or vegetables, because I know that's important to get more nutrients in your body. (Anni)*  *I gained loads of weight after having COVID, so now, I'm gonna have to lose weight seriously, because I know that it’s impacted my breathing. And I don't know how heavy I am because I can't stand on the scales. (Toby)*  *I think exercise and nutrition go hand in hand. I know when I'm exercising, my nutrition is good. If I have a period where I'm not exercising, my nutrition normally will go down with it. (Rosie)*  **Regulation of food intake for weight management purposes**  *I don't exercise, I'm not burning calories anymore. I've got weakness in my legs, so it's not good to put on weight. So, I must keep my weight down and eat as healthily as I can. (Drake)*  *I put pressure on myself, because I've put on weight since COVID, about four stone. I know that's not good for me. I also know the heavier I am, the more my muscles are going to have to carry. That could be making me tired. But I find it difficult to get enough calories so that I don't feel more tired but trying to be in a calorie deficit. Most people would build muscles and then burn more calories. But of course, I can't do that. I feel in that vicious circle, and I get cross with myself when I keep reaching for the cake and biscuits to give me that energy surge. But I can't get through the day, I cannot work unless I've got that energy from something. So, I feel in that vicious circle every single day. (Anni)*  *I always feel under pressure to eat healthy. Always, always, always. I’m always on a diet. Even in work, we sometimes do a group diet together. It’s tough to deal with. (Beck)*  *I would eat breakfast, but I try to keep my weight down and breakfast is the meal I miss the least. I can’t exercise anymore, so that’s my only option. (Flora)*  **Mindset**  *Part of healthy eating is your psyche and your motivation. If I didn’t have the will to cook, I would find it really distressing. (Harper)*  *It depends, what mindset I’m, whether I self-sabotage, which I do a lot. If something knocks me off, that can be it for weeks. (Joy)*  *It's always there, my awareness of healthy eating and how I should be eating healthy because it is good for me. But it gets shoved to the back of my mind depending on my mindset at the time. (Joy)*  *I'd like to eat healthy all the time. But I think sometimes, I don't know, situations, events, life just gets in the way. And you revert back to being very unhealthy. (Emmeline)*  *It's always been there the knowledge but it's doing something about it. Most of the time, it's hard to stick to it. I understand it takes dedication but obviously, convenience and sometimes work and life, you know, takeaways are easy. Ready meals are easy. I obviously understand that I need to eat healthier, but it's harder to do it than, saying I want to do it. (Dean)* |
| Physical capability | Physical skills  Physical capacity | **Physical symptoms = barriers to healthy eating**  *The physicality of food preparation. Chopping can be precarious, because it's a lot easier to chop my finger and carrying large pans of water for boiling. If my strength is not there, then that can be dangerous. (Grace)*  *Standing. I can't stand and prepare food for more than an hour. At the supermarket, I wear gloves, because it is so cold, I lose sensation in my fingers. (Harper)*  *The physical capabilities are tricky, the chopping, because otherwise, food can end up bouncing around the kitchen. (Joy)*  *I do experience that some things are more difficult to do, for example, I can’t stand in the kitchen very long. (Kim)*  **Budgeting of energy as fatigue management strategy**  *You have to factor in how tired you get and doing one job can throw you for the rest of the day. If you’re having a bad day, but it needs doing, then you'd only have so much energy to do everything else. It's difficult. You need to think, “How much energy is this going to take me and how tired am I going to be?” It’s a rubbish way to live. (Vera)*  *There are times when I experience tiredness, and it really does feel like proper fatigue. Luckily, it's not majorly often, but it does feel different to being a bit tired. I think if you're feeling that fatigue, you're less likely to want to get up and prepare a healthy meal from scratch, because I can see how it's so much easier to put a readymade lasagna or something in the microwave. (Grace)*  *It’s always managing energy levels. Doing what everybody else does normally in a day will drain my battery quicker. I have to budget my energy to make sure that I can finish what I start. (Drake)* |
| Physical opportunity | Environmental context  Resources | **Tools and adaptations to help overcome physical barriers to healthy eating**  *My hands are weak and they're a bit flawed. I have everything possible that can help me. I have food processers, spiralizers, things to open cans for me, things to beat things with. And I have everything nearby, so it's easy to use. (Kitty)*  *I have a seat in the kitchen. And that makes me more likely to cook from fresh. I sit on it and take my time. (Beck)*  *We recently got a new kitchen, and we deliberately designed it knowing that I would be less physically capable. (Anni)*  *I have a car, it's automatic. I can only drive automatic. I have a three-year licence, and I use it all the time. (Juliet)*  *I suppose we're lucky, we've got a car, I can get to the supermarket. (Emmeline)*  *I would say our number one thing is our air fryer. It's just changed the way we prepare meals. It's so easy to use, and it's less effort. But also what we've heard is it's better for you. So that is a big factor. Because you think, “I don't have to put so much effort into preparing this meal but it's still going to be good for me.” Which is obviously the ideal thing. (Grace)*  *You know, I’ve got blenders. Then I've got things that chop, to help me prepare food. Different attachments to various kitchen appliances that give me that nudge to actually make something healthy. (Gillian)*  *Because I like cooking, I got myself a good set of knives. That always helps me to do stuff. They're easier to chop things and I like doing the preparing with those. (Dean)*  **Challenges associated with online food shopping**  *They deliver huge baskets, and you've got to bend down. But I can only carry a couple of items at a time because of my balance, so it takes twice as long to do that. (Emmeline)*  *Trying to get shopping from the door into the kitchen. I know I should use online shopping more, but the number of times I've done it, and they've been out of stock of something, and substituted something quite major for something else, or the shelf life is bad. And once it’s at my house, I find it hard to tidy it away quickly. (Vera)*  **Privilege and (financial) resources**  *Cost is a huge thing, especially for disabled people and I recognise my privilege that I've got disposable income that I can choose to spend on food. (Tamsin)*  *Healthy food can be more expensive. Same with having fresh vegetables, salmon, and good quality meat. There's a cost associated with that. It's cheaper to eat less healthy food, generally. Having fresh fruit in the house all the time, that's a privilege. We're in a very luxurious position that I don't have to think about our food budget. So, it's not a barrier to me, but it is globally a barrier. (Kendra)*  *And you know, we have a nice kitchen and a dishwasher, all those nice things. So, we've got everything that we need. We're very privileged and lucky. (Kendra)I think also having the ability to buy good quality meat, fish, vegetables. I feel lucky to be in a position to be able to buy the good quality food. (Grace)*  *For some people, healthy eating is not as accessible as it is to me. You know, it is expensive. I would say things like that have an impact. I think for some people, that's just not something that they can afford to do. And in those scenarios, I can't say that it's as easy for me as it would be for anyone, I wouldn't say like anyone would be able to do exactly what I do, because I appreciate that there are probably cheaper ways to eat and that wouldn't be as healthy. (Rosie)*  *I suppose the issue with cost is I do use the oven quite a bit. And recently, of course, electricity or gas has gotten much more expensive. I think that's quite an issue, which is why people turn to microwave meals. (Harper)* |
| Psychological capability | Knowledge  Behaviour regulation  Cognitive skills  Memory, attention and decision processes  Social, emotional, and intellectual understanding | **Knowledge of healthy eating**  *Healthy eating is having a balance, knowing that you can enjoy the richer foods, foods that one would consider less good for you, sensibly of course. (Letty)*  *I'm very aware there's no one special diet for CMT. It’s just that boring, but healthy, balanced diet, you know, making sure you're getting a good spread of the things you need. (Kendra)*  *I've learned over the years that your stomach really does impact your brain quite a lot. There's real connections. I think because of that, I tend to make healthier choices, because I feel like if I ate something cheesy and carby, it would sit heavy, and I'd feel sluggish. I wouldn't want to go for a run as much and get all bloated, which is a horrible feeling. So, having an awareness of what's going to trigger a positive response to my body versus negative is useful. And it's a way that I can manage my well-being. (Grace)*  *I think you need to understand it, which I still don't in all honesty. I listen to other people and try it out and see how I feel. Maybe if there was a short course, or something that you could do in your own time, that taught you about nutrition and healthy eating. And, you know, I eat a lot of carbs because it gives me energy. But maybe that's not the right way that I should be eating and maybe that’s what’s making me more sluggish, because carbs got a lot of sugar in it. You know, I don't understand how to get energy without making myself feel sluggish. That would be interesting to learn. (Anni)*  *A couple of people have said to me that I should eat a lot of protein because I get it, in their head, protein builds muscle and then you're building muscle. But no one ever knows about muscle wastage conditions. So, it's not something that I can check. (Anni)*  **Planning and organisation**  *I plan ahead. The challenge for us is that my husband doesn't like cheese, so we use pulses, beans. I'll sometimes make lasagna and use lentils in the sauce and avoid putting cheese on top of it. I always have to make sure I have ingredients in the house to make those alternatives. (Harper)*  *If we're going out for a celebration meal with friends, because it's someone's birthday, the choice may be unhealthy then because it's a one off. Things that we do regularly, like when we do our bike rides regularly, we will take things, like a packed lunch, or have a look at the menu and go, “Actually, I could have that. That's quite good. There aren’t too many calories in there.” And plan to make healthy choices in that respect. (Emmeline)*  *I do a shopping list every week, and I will have planned pretty much all our meals, maybe not lunches, lunches tend to be three or four things that we would eat on rotation. And then our evening meal, I usually know what we're going to have. (Flora)*  *I'm gonna have tomato soup for lunch today. I checked before I came on, do I have enough fresh tomatoes in the garden to make some tomato soup? No, I don't. But fortunately, I made some before and it's in the freezer, so I've got it out. I planned it. I needed to get something ready to have a healthy lunch. I don't want to forget to get something out and then I'll have biscuits. I don't want to do that. (Jim)*  **Employment and healthy eating**  *Workdays are more challenging because I don't work from home. On workdays, I can't go into my kitchen and make something, like a salad, so I grab a sandwich normally, so I don’t have to prepare anything, which doesn’t have the vegetables in it that I would have in a salad. (Rosie)*  *I aim to cook from scratch when I finish work, but sometimes can't be bothered. So, I throw things in the oven or have pot noodles. I find it easier at the weekend. And Thursday. I don't work Thursday. On a Thursday I’m more inclined to do it, because sometimes I'll have nothing else to do that day. I think, “I'll make something from scratch” because that'll be my one thing for the day. (Beck)*  *I have my full-time days at the end of the week, so Wednesday, Thursday, Friday, it really starts dipping back to convenience foods. (Anni)*  **Retirement and healthy eating**  *Since I retired, I've got more time to think about eating. Which is good and bad. If I eat breakfast, it means I can’t eat any lunch because I don’t want to eat too much. So, it’s constantly weighing up my options. (Emmeline)*  *I try to manage what I eat, but I find that hard because of the snacking. It takes a lot of discipline. When I was working, it was much easier. I would take lunch to work. I didn't snack. (Letty)* |
| Social opportunity | Social influences  Reinforcement  Emotion | **Limited dietary support from healthcare professionals**  *Absolutely zero. I was in a teaching hospital, and they were kind but all they said was, “You've got this, and there's no cure.” Every time I go for a check-up, which is once a year, they are nice. It's the only time given me to talk about my symptoms and then they say, “Yes, this is normal”. But apart from it, nothing. (Kim)*  *It was just a case of “This is what you have, goodbye”. In my checkups with the neurologist now, no one has ever mentioned eating. (Emmeline)*  *I've had advice from the consultant. When I'm doing potatoes in a pan, because I'm cooking for a lot of us that means I need a big pan. And she suggested, “Why don't you put two smaller pans on?” Little things can make a huge difference. Or instead of straining the water out, use a spoon with holes to scoop out the potatoes. And you know what, those tips make a real positive impact. I still manage making nutritious healthy foods for the family. (Vera)*  **Support and information from (online) peer-groups and communities**  *The best thing about having CMT is the strong community. It encourages each other to be active and eat healthily. People need it. (Kendra)*  *I joined this support group in the West Midlands, they make me feel that help is out there. (Gillian)*  *It became easier when you could get information online. And when there were patient groups online. Because there's no cure, a lot of the things with CMT are practical things you can do to make life easier, and the best people to get advice on that, are people who are facing the same problems. You tend to see a doctor once a year, and they're busy, so you don't feel like you can ask many questions. (Flora)*  **Support from spouses and other family members**  *The preparation is a problem in my case, which is why my husband tends to cook. Bending down to put things in and out of the oven is difficult. I can prepare sometimes, but then my husband has to put it in the oven or take it out as it's not safe to get things out of the oven. It would end up everywhere. Chopping, I can chop most things, I’m still not too bad. As long as I haven't got tremors. Things like butternut squash or pumpkin, they are difficult. So, my husband usually cuts those up. And then I carry on after he opens tins for me or bottles, depending what it is and how I'm feeling on the day. I do quite enjoy cooking, but it's become a bit of a trial. (Emmeline)*  *In the evening, if I've been working all day, I'm quite tired. So, I might intend to cook and then say to my husband, “Oh, can you do it?” And he will. (Flora)*  *If I was living on my own, that would be a problem. I would still do online shopping but what's quite useful, if I forget something, my husband will pop to Tesco. It would be a big deal for me to have to do that. If he wasn't here, and he wasn't willing to help, then that would be difficult. I couldn't go to Tesco and carry a shop back. Definitely not. (Flora)*  *My husband can chop things for me. If he's around, he'll do it. (Beck)* |
